# Supplementary material for: Angelica sinensis polysaccharide as potential protectants against recurrent spontaneous abortion: focus on autophagy regulation
Source: Front Med (Lausanne). 2025 Jan 15;12:1522503. doi: 10.3389/fmed.2025.1522503 (PMC11774876; doi:10.3389/fmed.2025.1522503)
Supplement: Supplementary material S1 — The main instruments used during the LC-MS process, along with their models/specifications and manufacturers. [file Supplementary_file_1.zip › Metabolomics sequencing data FC1.2/8.代谢通路分析/Exposure_Ctrl/all/KEGG/mmu05152.html]

KEGG PATHWAY: Tuberculosis - Mus musculus (house mouse)


# Tuberculosis - Mus musculus (house mouse)


[
Pathway menu
| Organism menu
| Pathway entry
| Show description
| Download
| Help
]

Tuberculosis, or TB, is an infectious disease caused by Mycobacterium tuberculosis. One third of the world's population is thought to be infected with TB. About 90% of those infected result in latent infections, and about 10% of latent infections develop active diseases when their immune system is impaired due to the age, other diseases such as AIDS or exposure to immunosuppressive drugs. TB is transmitted through the air and primarily attacks the lungs, then it can spread by the circulatory system to other parts of body. Once TB bacilli have entered the host by the respiratory route and infected macrophages in the lungs, they interfere with phagosomal maturation, antigen presentation, apoptosis and host immune system to establish persistent or latent infection.


##### Option

Scale:


100%

Image resolution:


 High

##### Search

##### ID search

##### Color


KGML

Image (png) file 1x
